# A New Precision Minimally Invasive Method of Glial Scar Simulation in the Rat Spinal Cord Using Cryoapplication

**DOI:** 10.3389/fsurg.2021.607551

**Published:** 2021-07-15

**Authors:** Georgii B. Telegin, Alexey N. Minakov, Aleksandr S. Chernov, Vitaly A. Kazakov, Elena A. Kalabina, Vasily N. Manskikh, Dmitry S. Asyutin, Alexey A. Belogurov, Alexander G. Gabibov, Nikolay A. Konovalov, Aldo Spallone

**Affiliations:** ^1^Branch of Shemyakin and Ovchinnikov, Institute of Bioorganic Chemistry, Russian Academy of Sciences, Moscow, Russia; ^2^A.N. Belozersky Institute of Physico-Chemical Biology, M.V. Lomonosov Moscow State University, Moscow, Russia; ^3^Department of Spinal Neurosurgery, N.N. Burdenko National Scientific and Practical Center for Neurosurgery, RF Health Ministry, Moscow, Russia; ^4^Shemyakin and Ovchinnikov Institute of Bioorganic Chemistry, Russian Academy of Sciences, Moscow, Russia; ^5^Department of Clinical Neurosciences, NCL-Neuromed Institute of Neurosciences, Rome, Italy; ^6^Department of Nervous Diseases, RUDN University, Moscow, Russia

**Keywords:** rat, biomodel, glial scar, spinal cord injury, cryoapplication

## Abstract

According to the World Health Organization, every year worldwide up to 500,000 people suffer a spinal cord injury (SCI). Various animal biomodels are essential for searching for novel protocols and therapeutic approaches for SCI treatment. We have developed an original model of post-traumatic spinal cord glial scarring in rats through cryoapplication. With this method the low-temperature liquid nitrogen is used for the cryodestruction of the spinal cord tissue. Forty-five Sprague Dawley (SD) non-linear male rats of the Specific-pathogen-free (SPF) category were included in this experimental study. A Th13 unilateral hemilaminectomy was performed with dental burr using an operating microscope. A specifically designed cryogenic probe was applied to the spinal cord for one minute through the created bone defect. The animals were euthanized at different time points ranging from 1 to 60 days after cold-induced injury. Their Th12-L1 vertebrae with the injured spinal cord region were removed “e*n bloc*” for histological examination. Our data demonstrate that cryoapplication producing a topical cooling around−20°C, caused a highly standardized transmural lesion of the spinal cord in the dorsoventral direction. The lesion had an “hour-glass” shape on histological sections. During the entire study period (days 1-60 of the post-trauma period), the necrotic processes and the development of the glial scar (lesion evolution) were contained in the surgically approached vertebral space (Th13). Unlike other known experimental methods of SCI simulation (compression, contusion, etc.), the proposed technique is characterized by minimal invasiveness, high precision, and reproducibility. Also, histological findings, lesion size, and postoperative clinical course varied only slightly between different animals. An original design of the cryoprobe used in the study played a primary role in the achieving of these results. The spinal cord lesion's detailed functional morphology is described at different time points (1–60 days) after the produced cryoinjury. Also, changes in the number of macrophages at distinct time points, neoangiogenesis and the formation of the glial scar's fibrous component, including morphodynamic characteristics of its evolution, are analyzed. The proposed method of cryoapplication for inducing reproducible glial scars could facilitate a better understanding of the self-recovery processes in the damaged spinal cord. It would be evidently helpful for finding innovative approaches to the SCI treatment.

## Introduction

Spinal cord injury (SCI) is one of the leading causes of disability, associated with the inevitable formation of a glial scar in the post-traumatic period, which impedes the regenerative axonal growth through the lesion (1–3). An extensive search for potential therapeutic solutions has not provided promising results (4, 5), though some of the recently proposed therapeutic protocols, such as epidural stimulation, seem to be encouraging (6–8). Thus, it appears evident that reliable and easily reproducible animal models are needed to test testing perspective treatments.

Rodent models are typically used in the experiments simulating compression or contusion SCI (9–11). While these SCI models reproduce a realistic clinical course of the spinal cord injury in humans, they have multiple drawbacks, particularly, the impossibility to induce a “therapeutic” lesion. As a result, the glial scarring process in the experimental SCI simulation is associated with poor reproducibility, thus limiting the use of therapeutic treatments (treatment protocols) based on the received experimental data. Since post-traumatic glial scars either fully prevent the growth of axons through the lesion after SCI (12, 13), it seems essential and promising to find novel methods of SCI simulation that would ensure a high reproducibility of standardized parameters of the scar evolution. Previously we described a new precision and minimally invasive technique for producing a standardized glial scar in experimental rats based on the localized cryodestruction of the spinal cord (14). Here we assess the morphological characteristics of the glial scar and describe the stages of its formation using the proposed technique.

## Materials and Methods

### Laboratory Animals

Male SD rats of SPF-category (*n* = 45) weighing 320–360 g were used in the experiment to ensure an optimal visualization and identification of all anatomical structures, and provide adequate space for performing the surgical procedure. The animals were kept under standard housing conditions at the Research and Production Unit ≪Animal Breeding Facility≫ of the Branch of the Institute of Bioorganic Chemistry (BIBCh) of the Russian Academy of Sciences (Unique Research Unit ≪Biomodel≫ of the IBCh RAS accredited by the AAALACi). All manipulations with the animals were approved by the Institutional Animal Care and Use Committee of the BIBCh (Protocol No. 718/19 of 01.10.19).

### Preoperative Preparation and Anesthetic Support

The animals were placed in cages with clean bedding and water 24 h before the surgery. The weight of the animals was measured at the day of surgery. The surgical procedure was performed using a strictly controlled aseptic technique, under general anesthesia with Aerrane (Baxter Healthcare Corp., USA) on a heated operating table (+38°C). Premedication was not utilized. Body temperature and heart rate were monitored continuously during the surgery.

### Surgical Approach and Cryoapplication

The surgical technique was described previously (15, 16). Briefly, unilateral laminectomy of Th13 vertebra was performed using a dental drill with a diamond burr of 1 mm in diameter. Cryoinjury was induced with a thermally conductive assembly of the originally designed cryoprobe applied to the spinal cord through the dura mater. A copper conductor in the area of contact with the spinal cord was 0.8 mm in diameter, and the distance from the cooling source (liquid nitrogen) was 9 cm. The exposure to the cryoprobe lasted for 1 min. In the contact area the topical temperature reached −20°C.

### Postoperative Monitoring

Animals were euthanized at different time points following the cryoinjury: in the acute (first 24 h after the injury), subacute (days 3, 5, 7, 10, 14), and chronic (days 21, 30, 60) periods (five animals at each time point). The minimally invasive technique proposed for glial scar simulation maintained physiological functions of the animals in the postoperative period and allowed achieving their 100% survival rate.

### Assessment of the Locomotor Activity

Locomotor activity in rats was assessed in the preoperative period and daily during a 60-day post-injury period. Using a blinding approach, two independent investigators assessed the experimental groups of animals. They tested their locomotor functions according to the open-field locomotor rating scale described by Basso, Beattie and Bresnahan (BBB) (17). The tests were conducted in each of the animal groups (five rats per group) to assess the post-SCI recovery the hind limbs' locomotor functions. To perform the BBB test, each of the rats was individually placed in an open-field with a non-slip floor where the animal could move freely for 5 min. Two observers evaluated the movements of the animals in the open field and their ability to use the hind limbs according to the BBB locomotor rating scale, ranging from 0 (total absence of movements) to 21 (regular movements).

### Histological Methods

The topography of damaged structures of the spinal cord was analyzed using “A High-Resolution Anatomical Rat Atlas” (18). To improve visualization of the spinal cord lesion and assist with the preparation of histological sections, a methylene blue 0.1% solution (HiMedia Laboratories, India) was used. After the animals were euthanized and the spinal cord within the bone fragment was extracted, 100 μl of dye were added to the laminectomy window. Samples of the rat spinal cord encased in bone (the so-called “en masse”) corresponding to the length of three vertebrae (the vertebra of the surgical approach—Th13, and two adjacent vertebrae—Th12 and L1) were fixed in 10% neutral buffered formalin solution for 2–5 days, rinsed in running tap water and processed for decalcification in Trilon B at room temperature for 12–16 days. As soon as a satisfactory decalcification was achieved, the specimens were cut and cleaned of soft tissues. The biomaterial was oriented for further microtomy in the sagittal (four animals in each group) and frontal (one animal in each of the groups) planes. The cut specimens were rinsed in running tap water, dehydrated in an ascending alcohol series and embedded in paraffin. Serial sections were cut on microtome at 200 μm. Hematoxylin and eosin (H&E), Azan trichrome (for the selective identification of collagen fibers and glial elements), and Mallory's phosphotungstic acid hematoxylin (PTAH) (for detailed and selective staining of astrocytes) were used for serial 4–5-μm-thick paraffin-embedded sections. The sections were examined by the standard light microscopy with Axio Scope.A1 microscope (Carl Zeiss, Germany). The sagittal section with the largest lesion area and two adjacent sections in cranial and caudal directions were selected for morphological measurements. Photomicrographs of the histological sections were made with Axiocam 305 color high-speed camera (Carl Zeiss, Germany), and morphometric measurements were processed with ZEN 2.6 lite software (Carl Zeiss, Germany) and Image J, v.1.53e software (19). Morphometric methods and techniques were used to count the mean number of macrophages per unit area of the glial scar. It included measurements of the specific volume of newly formed blood vessels and fibrous scar tissue component in no <30 fields of vision on three sagittal sections. The specific volume of blood vessels and a fibrous component of the glial scar was expressed in mm^3^/mm^3^, which means the volume of a particular structure per mm^3^ of tissue. All data was present as mean +/- standard error.

## Results

### The Topography of the Lesion

**Figure 1:**
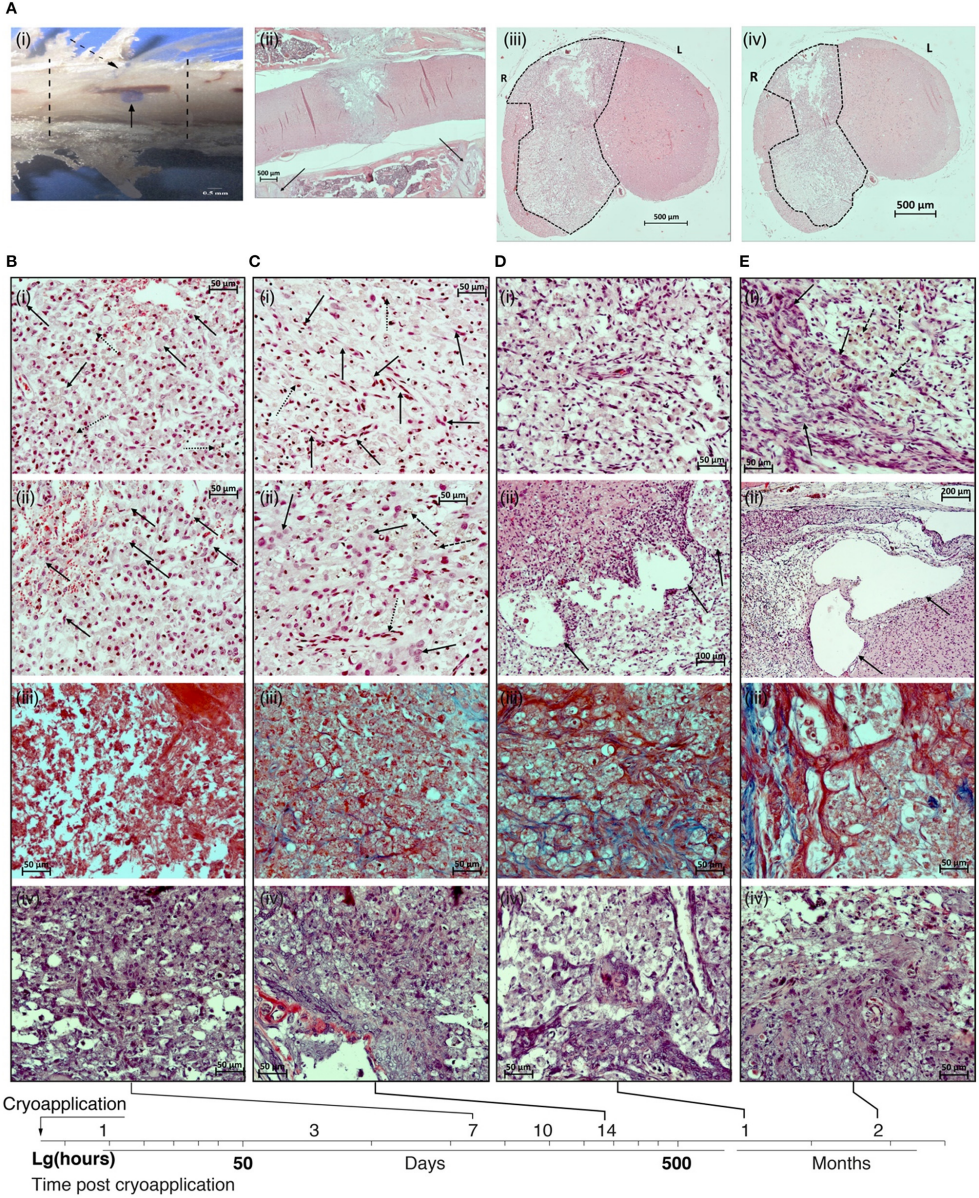
**(A)** Macro- and microscopic patterns of the spinal cord lesion. (i) sample of the spinal cord together with the surrounding bone tissue. Lesion of the spinal cord (solid arrow); cryoapplication zone in the dura mater (dotted arrow); margins of Th13 vertebra (dotted lines). (ii) Panoramic image of a sagittal section of the spinal cord in the vertebral column on day 21 after the cryoinjury. The lesion is transmural, localized at the projection of the Th13 vertebra, has an ≪hour-glass≫ shape. Projections of the margins of the vertebra used for the surgical approach are shown by solid lines. (iii) Panoramic image of the transversal section of the spinal cord in the rostral direction from the epicenter of cryoapplication at day 14 after the cryoinjury. Unilateral lesion. (iv) Panoramic image of the transversal section of the spinal cord in the caudal direction from the epicenter of cryoapplication at day 14 after the cryoinjury. Unilateral lesion. B-D: H&E staining; sites of formed lesions (dotted lines). 500 μm scale bar used for all images. **(B)** Photomicrographs of the lesion in the rat spinal cord at 7 day after the cryoinjury. (i) Fragment of the central part of the lesion at the projection of spinal gray matter: multiple macrophages transforming into ≪grainy spheres≫ (solid lines), numerous siderophages (dotted arrows) destroy the late-stage hemorrhagic component in the necrotic site; erythrophagocytosis process is in place. (ii) Fragment of the peripheral part of the necrotic site in the rat spinal cord adjacent to the intact tissue: multiple thin-walled blood vessels, proliferation of the endothelium (solid arrows). (iii) The absence of collagen fibers in the peripheral portion of the cryolesion. (iv) Reactive activation of astroglia at the projection of ventral funiculi of spinal white matter. **(C)** Photomicrographs of the lesion in the rat spinal cord at 14 day after the cryoinjury. (i) Fragment of the central portion of the lesion at the projection of spinal gray matter: a decreasing percentage of macrophages in the total population of cells in the lesion (dotted arrows). Multiple newly formed blood vessels (solid arrows). (ii) Fragment of the peripheral portion of the lesion: further decrease in percentage of macrophages in the total population of cells in the lesion (dashed arrows), the emergence of large glial cells (solid arrows). Newly formed blood vessels (dotted arrow). (iii) Emergence of collagen fibers in the glial scar (blue color). (iv) Reactive activation of astroglia at the projection of ventral funiculi of spinal white matter. **(D)** Photomicrographs of the lesion in the rat spinal cord at 30 day after the cryoinjury. (i) Fragment of the central portion of the lesion at the projection of spinal gray matter: multiple macrophages distributed throughout the lesion against the background of numerous newly formed blood vessels. (ii) Fragment of the peripheral portion of the lesion adjacent to the intact tissue: large cystic cavities (shown by arrows) at the interface between the lesion and the intact spinal cord. (iii) The percentage of collagen fibers (blue color) is increasing in the glial scar structure. (iv) Multiple astrocytes at the projection of ventral funiculi of spinal white matter. **(E)** Photomicrographs of the lesion in the rat spinal cord at 60 day after the cryoinjury. (i) Fragment of the peripheral portion of the lesion adjacent to the intact tissue: large cystic cavities at the interface between the lesion and the intact spinal cord. (ii) Fragment of the peripheral portion of the lesion: multiple macrophages (dotted arrows) against the background of glial cells and glial fibers (solid arrows). (iii) Mature collagen fibers (blue color) in the structure of glial scar. (iv) Multiple astrocytes at the projection of spinal gray matter. (i), (ii) – H&E, (iii) – Azan trichrome, (iv) – Mallory's phosphotungstic acid haematoxylin (PTAH) staining. Magnification 200 × (i–iv) except Dii (100×) and Eii (200×).

Macro- and microscopic patterns of the spinal cord lesion were studied and compared with the contralateral intact area. Macroscopically the injured area differed significantly from the non-exposed side of the spinal cord (Figure 1Ai) and was always localized at the surgical approach and contained within the Th13 vertebra.

In sagittal sections, the cryodestruction lesions were always transmural, that is, they extended throughout the dorsoventral dimension of the spinal cord, and beginning from the fifth day of the post-injury period the lesions acquired their final geometric pattern of an ≪hour-glass≫ shape, expanding from the dorsal side and narrowing from the ventral side. The narrowed portion of this “hour-glass” corresponds to the ventral third of spinal gray matter (Figure 1Aii). Frontal sections in the cranial and caudal directions from the epicenter of exposure to cryoprobe throughout the observation period demonstrated a unilateral lesion of the spinal cord—at the right side, exposed to cryoapplication (Figure 1Aiii,iv).

### The Acute Period After SCI

During the acute period (24 h after the cryoinjury), findings in the exposed area included tissue debris; massive microhemorrhages with a pronounced imbibition of the spinal cord tissues by fresh erythrocytes that could cause the expansion of necrotic zone in the first days of the post-injury period; few segmented neutrophils entering the necrotic area; neutrophil margination and diapedesis in the vessels adjacent to the necrotic area.

### The Subacute Period After SCI

In the early subacute stage (days 3–5 after the injury), the cryodestruction lesion was characterized by well-defined margins separating it from the surrounding intact tissue of the spinal cord. On day 3, the signs of hemorrhagic events were still present; both in the central and peripheral portions of the lesion, where the resorbed necrotized tissue was located, some clusters of segmented neutrophils were present. In general, on day 3 of the observation period, the necrotic area was characterized by hypocellularity. A pronounced vascular response was seen at the interface between the damaged and the intact spinal cord tissues. Individual macrophages, siderophages were visible at the lesion margins on the side of the intact tissue. Erythrophagocytosis was also observed. On day 3 of the post-injury period, the macrophage response at the peripheral portion of the lesion was only modest.

On day 5, the cryodestruction lesion was characterized by the so-called “inhibition of leucocyte infiltration”: a very few, if any, segmented leucocytes were present in the central portion of the lesion. Some segmented neutrophiles undergoing degradation were found. At the same time, the peripheral regions of the lesion appear to be largely infiltrated by macrophages. Some of them are beginning to transform into typically looking lipophage-like “grainy spheres” due to their lipid-rich cytoplasm composition. The maximum vascular response in the peripheral portion of the lesion was found at this time point. In addition, the initial manifestations of neoangiogenesis with an active role played by fibroblasts were evident at the peripheral portion of the lesion. Some signs of the proliferation of endothelial cells were also seen. Macrophages—transformed into siderophages—were involved in the active removal of the late-stage hemorrhagic components.

On day 7 after the cryoinjury, macrophages were distributed evenly throughout a larger area of the produced lesion in the spinal cord; their, highest activity was observed at the projection of spinal gray matter (Figure 1Bi,ii).

Usually, the lesion at the projection of spinal white matter appeared to be “hollow.” On day 7, the number of macrophages per area unit of the necrotic zone reached its peak: 68.1 ± 4.6 cells per field of vision of 37,500 μm^2^ (400 × magnification). Manifestations of erythrophagocytosis were seen both in the central and peripheral portions of the necrotic area, multiple siderophages also were found. In the central and peripheral parts of the lesion, erythrocytes were scarce and looked abnormally. High intensity of neoangiogenesis and an active proliferation of endothelial cells were seen at the margins of the necrotic zone. The average specific volume of the vessels in the lesion amounted to 0.0359 ± 0.0039 mm^3^/mm^3^. At this time point, a fibrous component was absent in the glial scar (Figure 1Biii); a reactive activation of astroglia was found in the peripheral portion of the lesion, at the projection of ventral funiculi of spinal white matter (Figure 1Biv).

In the late subacute stage (days 10–14), there was a gradual decrease in the number of macrophages in the lesion cellularity. In parallel, glial cells were emerging, the process of neoangiogenesis was progressing, and the proportion of collagen fibers in the scar tissue was growing. As a rule, macrophages formed clusters suggesting that the phases of gliomesodermal scarring varied in different regions of the lesion. The newly formed vessels were found in the central part of the lesion and their specific volume increased considerably, from 0.0359 ± 0.0039 to 0.0504 ± 0.0021 mm^3^/mm^3^. The histological structure of the vascular walls was gradually returning to its regular pattern.

On day 14 after the cryoinjury, the macrophage percentage of the total cell population was still decreasing in the lesion (to 43.2 ± 3.9 cells per field of vision of 37,500 μm^2^); the newly emerged multiple clusters of glial cells were spread unevenly over the lesion. The scar tissue was characterized by a regular vascularization pattern and the blood vessels returned to an ordinary histological organization (Figure 1Ci,ii). By day 14, the mean specific volume of blood vessels in the lesion increased considerably and reached 0.0725 ± 0.0044 mm/mm^3^. The specific volume of collagen fibers in the glial scar structure reached 0.0718 ± 0.0041 mm^3^/mm^3^ (Figure 1Ciii). A pronounced reactivity of astroglia was found in the peripheral portion of the lesion, at the projection of ventral funiculi of spinal white matter (Figure 1Civ).

On day 21 of the observation period, the number of macrophages was still high. They were distributed evenly throughout the formed lesion in the spinal cord (to 42.7 ± 4.2 cells per field of vision of 37,500 μm^2^). However, their morphology and size varied within the same histological sections (the variation could correlate with the time of presence of the cells in the cryoinjury-caused lesion). Clusters of glial cells were seen in the peripheral portion of the lesion. The share of the fibrous tissue component in the structure of the gliomesodermal scar was noticeably increasing as compared to day 14. At the same time, the artificial hollow spaces in the sections were still present. It is worth noting that 3 weeks after the exposure, a trend in the development of cystic cavities was observed at the site of cryoapplication. Multiple newly formed blood vessels with an ordinary histological structure were visible all over the area. The average specific volume of blood vessels in the lesion reached 0.0807 ± 0.0037 mm3/mm^3^. The specific volume of collagen fibers in the glial scar structure reached 0.0826 ± 0.0034 mm^3^/mm^3^.

### The Chronic Period After SCI

By day 30 of the observation period, multiple macrophages were still present and distributed evenly throughout the lesion in the spinal cord (to 45.8 ± 3.5 cells per field of vision of 37,500 μm^2^), while their morphology and size varied within the same histological section (Figure 1Di). The number of artificial hollow spaces was markedly less than at days 14 and 21 after the cryoinjury (Figure 1Dii). The share of the fibrous tissue component in the structure of gliomesodermal scar was increasing and reached 0.0932 ± 0.0036 mm^3^/mm^3^ (Figure 1Diii). Multiple newly formed blood vessels with a typical histological structure were spread over the examined areas. The average specific volume of blood vessels in the lesion reached 0.0878 ± 0.0013 mm^3^/mm^3^. Reactive activation of astroglia was seen in the peripheral portion of the lesion at the projection of ventral funiculi of spinal white matter (Figure 1Div).

Two months after the cryoinjury, in all animals the spinal cord lesion included large cystic cavities (up to 0.8 mm^2^) containing an amorphous substance (Figure 1Ei). Non-homogeneous, moderate cellularity with prevailing macrophages was seen in the lesion; multiple macrophages were still present, though they were not found in a few fields of vision (to 21.3 ± 2.7 cells per field of vision of 37,500 μm ^2^) (Figure 1Eii). Vascular network in the spinal cord lesion is developed, blood vessels have a typical histological structure. By the end of month 2 of the observation period, a mean specific volume of blood vessels in the lesion reached 0.0881 ± 0.0024 mm^3^/mm^3^. The fibrous tissue component was poorly represented in the structure of the gliomesodermal scar, the fibers were unevenly distributed over the section and undergone different stages of maturity (Figure 1Eiii). The specific volume of collagen fibers reached 0.1218 ± 0.0027 mm^3^/mm^3^ in the structure of the gliomesodermal scar. Glial cells and fibers were concentrated at the lesion margin (Figure 1Eiv). Thus, by day 60 of the observation period, all findings showed that glial scar structures stabilized and that a mature fibrous component was formed.

**Figure 2:**
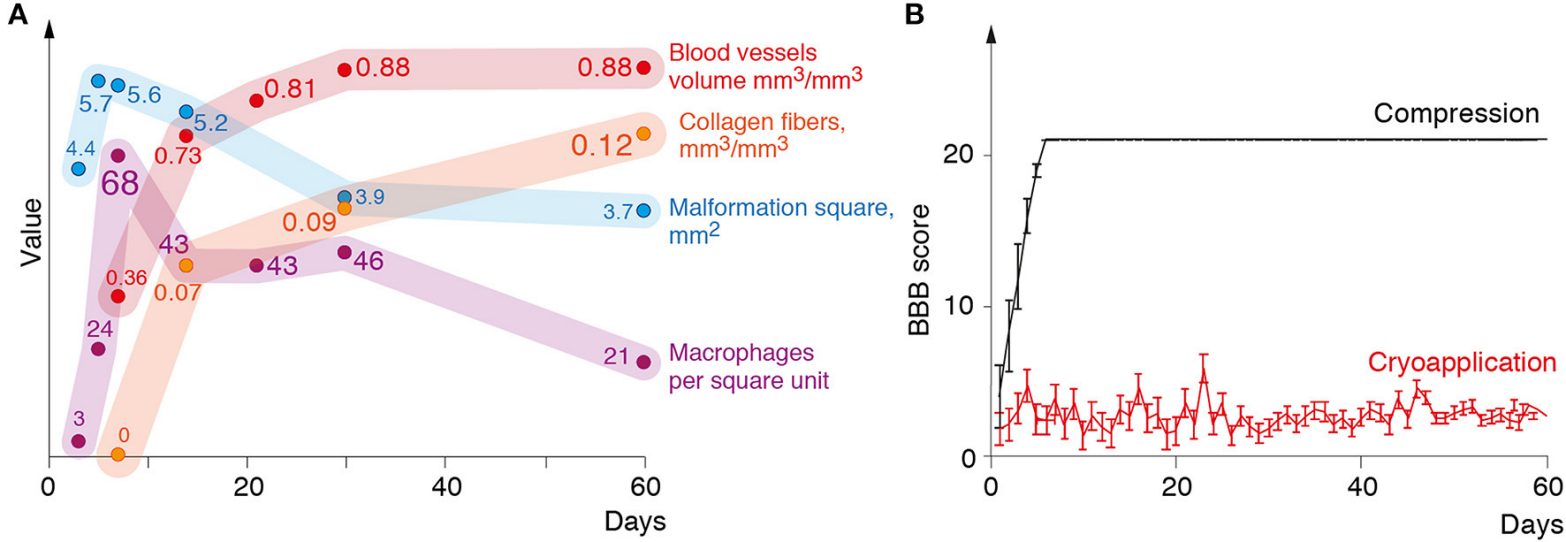
**(A)** Changes in the lesion size, the number of macrophages, volume of blood vessels, and connective tissue in the lesion during 60 days following the cryoinjury. **(B)** Mean BBB (Y-axis) score of the rats over a 2-month observation period (X-axis).

Figure 2A illustrates morphometric measurements of the lesion size, number of macrophages, specific volume of blood vessels and collagen fibers in the structure of glial scar at different time points after the cryoinjury.

The impact of the cryoapplication on locomotor activity of rats was tested in an open field according to 21-score BBB Locomotor Rating Scale. Animals from the experimental group after the cryoinjury had stable monoplegia with persistent disorders of locomotor functions at a mean 2.7 BBB score from day 1 through day 60 (Figure 2B). In surgical sham controls where the only surgical approach to the spinal cord was performed with the compression induced by a probe (without cryoinjury), the full recovery of locomotor activity was recorded much earlier—by day 5 after the surgery, as proven by BBB Locomotor Rating Scale (Figure 2B).

## Discussion

The histological studies have provided verifiable data on the timeline of glial scar formation following a standardized cryoinjury of the rat spinal cord. In general, the observed scarring process showed an expected sequence of events. We demonstrated that the lesions caused by the application of cryoprobe were transmural in serial sagittal sections, that is, they extended through the dorsoventral dimension of the spinal cord along its entire length.

Formation of an ≪hourglass-shaped≫ spinal cord lesion (which appeared to be highly reproducible in multiple animals) with tissue necrosis was observed in the acute post-injury period. The spinal cord lesion induced by cryoapplication extended to the right dorsal, right lateral, and right ventral funiculi of the spinal white matter, as well as the right dorsal and ventral horns of spinal gray matter. It ensured the development of a stable monoplegia, which was not followed by self-recovery, on the other hand—the absence of critical complications. It is worth noting that the commonly used methods of SCI simulation do not meet this requirement (11, 20). Currently used SCI models, in which self-recovery processes do not affect research findings, cause a severe dysfunction of the urinary system in rats, which is considered as a major complication (20), because it is necessary to empty manually the bladder of the animals several times a day after the injury to avoid bladder rupture and infection (21, 22). Our proposed method was free of such drawbacks due to the minimal size of the produced injury. The animals maintained the ability to naturally empty their bladder and intestines during the entire observation period despite the persistent monoplegia.

The subacute period was characterized by the macrophage-mediated reabsorption of necrotic tissue. This process was followed by localized neoangiogenesis and ultimately resulted in the development of mesodermal-glial scarring tissue and the formation of cysts. For the first time, macrophages appeared in the peripheral portion of the lesion on day 3 and demonstrated exponential growth by day 7. However, their number reduced by day 14 and reached a plateau by the end of the first month. It should be pointed out that at day 60 multiple macrophages were still present in the lesion. These findings prove that the rearrangement processes occur continuously in the course of glial scar formation (12, 23). Beginning from day 14, connective tissue was clearly visualized in the lesion. The fibrous component was increasing up to day 60. Similar changes are commonly found in humans with SCI (12).

As known, the glial scar is formed as a result of the reactive response of astrocytes after CNS injury (24). These reactive astrocytes migrate toward the epicenter of the lesion and have an impact on the process of tissue recovery. However, ultimately they turn into scar astrocytes and form s glial scar, which produces axonal growth inhibitors and prevents axonal regeneration (25, 26). We demonstrated that after the cryoinjury, the reactive astroglia was present in the lesion as early as day 7 and remained there up to day 60. It is highly likely that our method proposed for inducing a glial scar, reproduces the same pathological condition in humans (24).

Neoangiogenesis in the peripheral portion of the lesion was already recorded on day 5. The newly formed blood vessels begin to acquire typical histological structure by the day 14 of the observation period; their specific volume demonstrates an exponential progression and reaches a plateau by the day 30. Adequate nutrition of the forming scar is crucial at the early stages. But it is equally important that the newly formed blood vessels keep functioning at the late stages of maturation of the glial scar (12, 27). In our opinion, quantitative and especially qualitative indicators of the pass-through capacity of newly formed blood vessels may be considered as critical predictors of the effectiveness of repair processes in nerve tissue. To this end, our future research will be focused on neoangiogenesis and characterization of vascular networks in the scar tissue. Thus, the proposed model can be used as an efficient and reliable experimental simulation method of glial scarring in the post-traumatic period. It is also noteworthy that the spinal cord lesions were usually characterized by high reproducibility and low interanimal variability. According to the BBB score, all rats developed stable monoplegia at the injured side, which persisted for 60 days. In this context, the proposed model looks even more promising. However, this model does not represent the scenario of pathophysiological events in human SCI.

As a rule, for the reproduction of clinical cases, researchers use other well-known experimental SCI models. Nevertheless, we would like to re-emphasize that this model is focused on the issue of post-traumatic glial scarring, one of the most critical consequences of spinal trauma, for the management of which no successful therapeutic solutions have been found so far (28–30). The model proposed in this study offers considerable advantages compared to similar models of milder (compression and contusion) and more severe (partial/full interruption) injuries. It is known that self-recovery processes are more common for the models of milder SCI. Therefore, they seem to be inappropriate for the therapeutic screening (11, 15, 20). The proposed model falls in between an invasive contusion injury and the complete interruption of the spinal cord. The experimental rats maintained all the physiological functions, including the ability to empty their bladder and intestines naturally. Still, the same time, the self-recovery of locomotor activity did not occur at the side of the injury. Basically, a non-invasive cryoinjury is similar to a non-invasive contusion trauma which includes some of the spinal cord injuries in humans (31, 32). As a result, the spinal meninges, blood vessels, and cell processes were not damaged. Moreover, it was possible to avoid a rough physical disintegration of the rostral and caudal segments. The integrity of spinal meninges in our model enables us to create a more benign lesion considered as another substantial advantage. On the contrary, a decompression durotomy followed by the restoration of the meninges will reduce the formation of scars and decrease the size of the lesion (33).

Thus, the proposed glial scar model could serve as an optimal platform for assessing the effectiveness of potential therapeutic solutions targeted at the formation of glial scar in the post-injury period and searching for approaches to restore the conduction function of the spinal cord.

## Data Availability Statement

The raw data supporting the conclusions of this article will be made available by the authors, without undue reservation.

## Ethics Statement

The animal study was reviewed and approved by Institutional Animal Care and Use Committee of the BIBCh (Protocol n. 718/19 of 01/10/19).

## Author Contributions

GT, AS, AB, and AG: conceived of the presented idea. GT, VK, and AC: developed the theory and performed the data analysis. EK, VK, VM, DA, and AM: verified the analytical methods. AG, AS, and NK: supervised the findings of this work. All authors discussed the results and accepted the final version of the manuscript.

## Conflict of Interest

The authors declare that the research was conducted in the absence of any commercial or financial relationships that could be construed as a potential conflict of interest.
